# Logical fallacies persist in invasion biology and blaming the messengers will not improve accountability in this field: a response to Frank et al.

**DOI:** 10.1007/s10539-023-09892-3

**Published:** 2023-01-18

**Authors:** Radu Cornel Guiaşu, Christopher W. Tindale

**Affiliations:** 1 Biology Program, Glendon College, York University, 2275 Bayview Avenue, Toronto, ON M4N 3M6 Canada; 2 Department of Philosophy, University of Windsor, 401 Sunset Ave., Windsor, ON N9B 3P4 Canada

**Keywords:** Invasive species, Logical fallacies, Native range, Invasion biology, Non-native species, Precautionary principle

## Abstract

We analyze the “Logical fallacies and reasonable debates in invasion biology: a response to Guiaşu and Tindale” article by Frank et al., and also discuss this work in the context of recent intense debates in invasion biology, and reactions by leading invasion biologists to critics of aspects of their field. While we acknowledge the attempt by Frank et al., at least in the second half of their paper, to take into account more diverse points of view about non-native species and their complex roles in ecosystems, we also find the accusations of misrepresenting invasion biology, for instance by “cherry-picking” and “constructing ‘straw people’”, directed at the Guiaşu and Tindale study to be unwarranted. Despite the sometimes harsh responses by leading invasion biologists to critics of their field, we believe that persistent and fundamental problems remain in invasion biology, and we discuss some of these problems in this article. Failing to recognize these problems, and simply dismissing or minimizing legitimate criticisms, will not advance the cause, or enhance the general appeal, of invasion biology and will prevent meaningful progress in understanding the multiple contributions non-native species can bring to various ecosystems worldwide. We recommend taking a more open-minded and pragmatic approach towards non-native species and the novel ecosystems they are an integral part of.

## Introduction

We acknowledge the attempt by Frank et al. (2019) to analyze and respond to the Guiaşu and Tindale (2018) article, and we appreciate the interest shown by Frank et al. (2019) in this work. Unfortunately, while the paper by Frank et al. (2019) does try to take a more moderate and nuanced approach towards critics and criticisms of aspects of invasion biology, we still find that these authors appear to misunderstand and misrepresent certain sections of the Guiaşu and Tindale (2018) article, while ignoring other sections altogether. As a result, we think this response to Frank et al. (2019) is useful in clarifying our position on the issues under consideration while illustrating clearly some of the areas of continued disagreement resulting from different perspectives on non-native species and their complex roles in ecosystems. We believe this continued debate is important since it concerns the way we perceive and treat the multitude of non-native species found in various ecosystems and the way we relate to the natural world and choose to allocate the limited resources available for conservation programs, as well as the ways we use reason in such debates.

## Logical fallacies and continuing debates about invasion biology

### The familiar accusation of “cherry-picking”

In their response to the Guiaşu and Tindale (2018) article, Frank et al. (2019) resort to the often-used accusation of “cherry-picking”—a familiar tactic employed by some invasion biologists and their allies (for example, Hiltner 2018; Ricciardi and Ryan 2018b; Courchamp et al. 2020; Frank 2021) against critics of their field. We find this accusation to be often unwarranted. The accusation of cherry-picking seems to be made by some invasion biologists when they encounter inconvenient facts and examples they would rather not address (Guiaşu 2016; Pereyra and Guiaşu 2020). Guiaşu and Tindale (2018) cited 80 relevant articles and books, in a thoroughly researched and comprehensive paper. One can always accuse almost every author of a scholarly work, including invasion biologists, of “cherry-picking” sources, since there are only so many references that can be used in each case, and only so many topics that can be addressed and examples that can be included in any one study, and also because of the specific maximum word counts and numbers of pages allowed for particular categories of peer-reviewed articles in various journals. It is relatively easy to disagree with the selection of sources or examples and suggest alternative ones, but we think that Guiaşu and Tindale (2018) supported their arguments with an abundant, appropriate and representative set of references cited accurately and in the proper context. Numerous other relevant examples and case studies were also discussed in the detailed book by Guiaşu (2016) which is cited and referred to in both the Guiaşu and Tindale (2018) article and the Frank et al. (2019) response to that article.

### Guilt by association

Guilt by association remains a problem in invasion biology, as discussed by Guiaşu and Tindale (2018). An examination of the introductory sections of numerous articles in this field, about various non-native species, reveals that these articles typically start with statements about the negative role of, and the costs associated with, introduced species in general, and often a few notorious invaders, unrelated to the main species discussed in these articles, are also mentioned as examples of just how “bad” invasive species can be (Thompson 2014; Guiaşu 2016; Guiaşu and Tindale 2018).

It should be pointed out that even some of these supposedly very bad invaders do not turn out to have such negative effects overall after additional scrutiny and after more comprehensive studies of their impact are conducted. For example, a thorough review of the scientific literature on purple loosestrife (*Lythrum salicaria*) revealed that this much maligned introduced wetland plant, originally from Europe, does not reduce biodiversity in North American wetlands after all and can provide useful resources for a variety of native animals on this continent (Guiaşu 2016). Similarly, unfairly negative portrayals of the ecological roles of other non-native species were also analyzed and revealed in the scientific literature. Examples include the *Tamarix* shrub introduced to western areas of the U.S.A. (Stromberg et al. 2009), the European starling (*Sturnus vulgaris*) in North America (Koenig 2003), and the rusty crayfish (*Faxonius rusticus*) (Guiaşu and Labib 2021). The latter example will be analyzed in more detail in the next section of this article.

In addition, as discussed by Guiaşu (2016), alarmist general statements about introduced species continue to appear in the invasion biology literature even after these statements have been thoroughly debunked. For example, the commonly made and widely cited assertion that invasive species represent the second most important global threat to native species and biodiversity was debunked by Mark Davis (2009), in his comprehensive *Invasion Biology* textbook, but that did not stop Luque et al. (2014) from repeating, at the very beginning of the Introduction section of their article, that “invasive alien species (IAS) are regarded as the second largest threat to biodiversity worldwide”. The article by Luque et al. (2014) had seven authors, including Daniel Simberloff—the Editor-in-Chief of the flagship journal *Biological Invasions*. This article focused on a new addition to the rather subjective IUCN list of 100 of the world’s worst invasive species. Since then, the shaky foundation of this sweeping and very influential allegation against invasive species was also thoroughly analyzed and exposed by Guiaşu (2016) and, in great detail, by Chew (2015).

The often-repeated general anti-invasive species statements, and the frequent use of the same examples of well-known invasive species even in studies that focus on completely different species, do lead to a negative perception of non-native species in general, and therefore assign “guilt by association” to introduced species that may not have proven negative effects.

## Crayfish and fish case studies

We find it surprising that Frank et al. (2019) chose to focus on the rusty crayfish example discussed by Guiaşu and Tindale (2018), since, as far as we are aware, neither Frank nor the other co-authors of the Frank et al. (2019) article have ever worked with crayfish anywhere. On the other hand, one of us (Radu Guiaşu) has conducted crayfish studies in the field, the laboratory, and museum collections since 1991. Based on these more than thirty years of directly relevant experience, and a thorough recent review of 430 studies published between 1852 and 2018 (Guiaşu and Labib 2021), which mention the rusty crayfish, we can state confidently that the native range of this crayfish species is not well defined, and uncertainties persist about the history and extent of this range. It is unclear whether the rusty crayfish is native or non-native in several North American regions and jurisdictions, including parts of Ontario. The historical museum records examined for this species in Ontario are very scarce and incomplete, and do not provide a solid foundation for any reliable conclusions about the history of this crayfish in this province (Guiaşu 2016). In fact, the concept of native range, which is fundamental in invasion biology, is not well defined, and therefore not universal, and not useful in many cases, as discussed by Pereyra (2020) and Pereyra and Guiaşu (2020). Without a clear understanding of the history and limits of the native ranges of quite a few species, we obviously cannot determine their native or non-native status at many locations.

Frank et al. (2019) then proceeded to venture further into this discussion by referring to the impact of the rusty crayfish in Ontario and bringing up the Edwards et al. (2009) study. That study, which was based on a major survey of 100 lakes from a large region of south-central Ontario, found significant and geographically widespread population declines for all seven crayfish species (native and non-native) sampled, including *Faxonius rusticus* (previously known as *Orconectes rusticus*), commonly referred to as the rusty crayfish. This major decline, which occurred since the early 1990s, resulted in reduced crayfish species diversity and significantly lower, or even lost, populations of all crayfish species in this vast region. The rusty crayfish was indeed rare in this region, but local populations of this species declined by 91% during this period of time. This suggests that similar changing environmental factors, such as possibly reduced calcium concentrations in lakes, are negatively affecting all crayfish species in this region, regardless of their perceived native or non-native status. Edwards et al. (2009) wrote that they “found no evidence to indicate that the presence of nonnative crayfish was related to the decline of native species” and that the supposedly invasive rusty crayfish “was not a factor in the declines” of these crayfish species in Ontario. Therefore, despite the objections by Frank et al. (2019), the reporting of these findings in the Guiaşu and Tindale (2018) article was entirely accurate and relevant. The point Guiaşu and Tindale (2018) were making was that declines in crayfish species abundance and distribution can occur for a variety of reasons, and the rusty crayfish cannot and should not always be blamed, especially without proper evidence.

In fact, the impact of the rusty crayfish on other species and freshwater ecosystems can be complex, and is not nearly as straightforward as Frank et al. (2019) suggested. Guiaşu (2016) discussed this topic in a fair amount of detail in his book on non-native species, and showed, for example, how sometimes inaccurate citations and sloppy citation practices can lead to unwarranted extrapolations about the supposedly negative role of the rusty crayfish in regions where this role had not been directly investigated. For example, information about the rusty crayfish collected in some northern Wisconsin lakes was then automatically extended to other regions, including Ontario, where this species was present, without any direct evidence gathered in these other regions. The bad reputation acquired by the rusty crayfish in North America appears to be based mainly on studies from these northern Wisconsin lakes. A thorough analysis of the relevant literature reveals that our current understanding of the native range and the impact of this North American crayfish in its home continent is still quite incomplete, and the negative views of the ecological role of this species may be shaped more by the perception of *F. rusticus* as an invasive species than by an objective comparison to other similar crayfish perceived as native in various regions (Guiaşu, 2016; Guiaşu and Labib 2021). In addition, the rusty crayfish can also have positive effects, for example as an effective predator of the invasive zebra mussel (*Dreissena polymorpha*) in North America (Perry et al. 2000).

Frank et al. (2019) then stated that, in the 2018 article: “Guiaşu and Tindale also fail to mention the major cause of crayfish decline that Edwards et al. (2009) identified, introduced predatory smallmouth bass.” However, despite this categorical statement, it should be noted that Edwards et al. (2009) actually stated quite clearly: “We hypothesize that the introduction of predatory smallmouth bass (*Micropterus dolomieui*), increases in Al concentrations, and reduced Ca concentrations in these lakes are negatively affecting crayfish populations.” Thus, the effect of smallmouth bass mentioned in this study was based on a hypothesis, rather than an established fact-based conclusion. Furthermore, it could be argued that Frank et al. (2019) cherry-picked the possible effect of smallmouth bass as “the major cause of crayfish decline”, and ignored the other water chemistry-related factors, because the former fits their preferred narrative while the latter two do not.

In addition, just as in the case of the rusty crayfish, the exact limits of the native range of the smallmouth bass are unclear, and this fish species is actually native to parts of Ontario, and may have expanded its range naturally (in other words, entirely on its own), and/or with human assistance to other parts of this province. In their monumental classic book on the freshwater fishes of Canada, which remains the most comprehensive work of its kind, Scott and Crossman (1973) mentioned that the “original range” of this fish species included “the Great Lakes-St. Lawrence system (apparently excluding Lake Nipigon but including nearby lakes tributary to the Nipigon River and the north shore of Lake Superior)”. Scott and Crossman (1973) also mentioned that this fish species was first introduced outside what they call its “natural range” in Ontario as early as 1901. This raises the familiar question: for how long does a species have to exist at a particular location, before we can accept it as a “natural” part of local ecosystems, and stop treating it as an unwelcome intruder? Would 121 years be sufficient?

In a much more recent guide to the freshwater fishes of Ontario, Holm et al. (2009) stated that the smallmouth bass is “native to southern Ontario” but “has been widely introduced throughout central Ontario”. However, based on the distribution map given for the smallmouth bass in the Holm et al. (2009) book, and the map of the study area shown in the Edwards et al. (2009) article, it appears that most, if not all, of the lakes analyzed by Edwards et al. (2009) fall within the native range of the smallmouth bass. Therefore, according to this information, the smallmouth bass would not really be an introduced species in this region. Furthermore, the supposedly native and non-native ranges of the smallmouth bass in Ontario are adjacent to each other, and there is no clear geographic barrier between them. Therefore, this fish species can easily expand its range without human help throughout various interconnected freshwater bodies of water in Ontario which provide suitable environmental conditions. So, as in many other cases, the boundary between the native and the non-native ranges of this species is quite arbitrary, and we don’t really know exactly how, or when, the smallmouth bass, or the rusty crayfish, reached certain parts of Ontario that are assumed to be outside their “natural” range. We suspect that if the smallmouth bass and the rusty crayfish would be considered native in some areas where they are currently regarded as introduced (even though they may well, in fact, be native), all the talk about their presumably negative impacts would disappear, and their ecological roles would be reassessed and seen as perfectly “natural”.

For example, Guiaşu (2002, 2016), Guiaşu et al. (1996a, b), Guiaşu and Dunham (1999a, b), and Guiaşu and Guiaşu (2003) studied and analyzed the ecology, life histories, distributions, and aggressive behaviors of the two surface waters *Cambarus* crayfish species of Ontario and found that one of these two species (*C*. *robustus*) dominates the other one (*C*. *bartonii*) in interspecific agonistic contests. The two species have similar habitat requirements and life cycles, and the dominant species, *C*. *robustus*, appears to have expanded its range in Ontario, possibly at the expense of *C*. *bartonii*. It is possible that *C*. *robustus* may competitively exclude *C*. *bartonii* from some locations where the ranges of these two species overlap. Because both of these crayfish species are considered native in Ontario, the apparent displacement of *C*. *bartonii* by *C*. *robustus* from some regions of this province was not of concern, and was considered perfectly natural. However, if a crayfish species suspected of being non-native, such as *F*. *rusticus*, expands its range, in Ontario or elsewhere, conservation and invasion biologists interested in crayfish consider this to be a negative development, and advocate control and eradication programs to stop “the invasion” (for example, Hamr 2010). This is a clear double standard, particularly since all crayfish species currently present in Ontario have arrived since the end of the last Ice Age, their means of dispersal are unknown, and the oldest museum records available for these species in this province are from the early 1900s (Guiaşu et al. 1996a; Guiaşu 2016; Guiaşu and Labib 2021). Therefore, we know nothing about the distributions of these crayfish species in Ontario before the early twentieth century, and we simply do not have enough information to make definitive judgements about which of these species may be “native” or “non-native”. Our concerns about the ecological impacts of these crayfish are strongly linked to, and influenced by, the biogeographic labels we arbitrarily chose to attach to them.

## Attitudes towards non-native species in invasion biology

### “Guilty until proven innocent” approach revisited

Frank et al. (2019) claimed that Guiaşu and Tindale (2018) misrepresented the position of invasion biologists with regard to the so-called “precautionary principle” as applied to non-native species. In fact, this is a repeat of a similar accusation Frank (2021) previously directed at the Guiaşu and Tindale (2018) study, and basically the same defense of the general attitudes of invasion biologists towards non-native species was made several times before by Simberloff (for example, in 2003a and 2009)—the second author of the Frank et al. (2019) article—in response to other critics of aspects of invasion biology. The article by Frank appeared in print in 2021, but was actually published online in 2019, prior to the publication of the Frank et al. (2019) response. In this context, the name “precautionary principle” is a nicer label, or a euphemism, for the “guilty until proven innocent” philosophy of invasion biology, as described, for instance, by Ruesink et al. (1995) and Simberloff (2003a, 2007). The general idea seems to be that non-native species should be considered “guilty until proven innocent”, since they may eventually have negative effects on the ecosystems where they have been introduced, and, therefore, as a precautionary measure, these species should be controlled or eradicated, even in the absence of evidence that they are harmful.

The problem, however, is not with the perfectly accurate and logical way in which Guiaşu and Tindale (2018) analyzed the “guilty until proven innocent” approach towards non-native species promoted by invasion biology. The real problem is that some leading invasion biologists, such as Simberloff (2003a, 2009), take contradictory positions on this issue, sometimes within the pages of the same article. On the one hand, for example, Simberloff (2003a, 2009) claims that invasion biologists are not against all non-native species, and are only against the introduced species with negative impacts, but, on the other hand, he states that non-native species can become harmful at some unspecified point in time in the future, even if they are not harmful now, and, therefore, one should always be suspicious of non-native species in general, just in case. This is a clear contradiction. To put it simply, the position of these invasion biologists seems to be: no, we are not against all non-native species, just the bad ones, but they may all become bad one day, so let us treat them all as potentially guilty essentially forever. How is this position not prejudicial towards all non-native species? The position of leading invasion biologists on this issue is unambiguously illustrated by the following definitive statement: “All introduced species must be considered potential invaders, since many lie dormant for years or decades, starting to invade and cause damage only when certain conditions for reproduction or spread are realized.” This statement was made by Richardson et al. (2008) in an article with five authors, including Simberloff. So, to these invasion biologists, a non-native species is either harmful or potentially harmful.

When legitimately criticized for the obviously negative attitudes towards non-native species their field is based on, such invasion biologists retreat to the position that they do not dislike all non-native species, just the few with negative effects on ecosystems. But in fact, the application of their own version of the “precautionary principle” means that they generally regard non-native species as problematic or potentially problematic from the start, and monitor these species looking for harmful effects. And even if such harmful effects cannot be found, non-native species can never be fully exonerated, since the harmful effects may not become apparent until later, at an undetermined point in the future, possibly centuries from now. Can we really make conservation plans now, and take action against species without any proven negative impacts, just in case some of them may turn “bad” somehow centuries from now? Is this a realistic conservation goal that should be seriously and urgently debated at this time? Don’t we have more pressing and more immediate environmental concerns and priorities? Many environmental changes, quite a few of them undoubtedly hard or impossible to predict with great accuracy, will occur during the next few centuries, and trying to anticipate now which species may cause problems hundreds of years into the future seems like an impractical pursuit.

This problem is even more challenging, because invasion biology is not good at generating useful predictions about which non-native species are most likely to have negative effects—a fact acknowledged even by several researchers in this field (for example, Simberloff 2009; Kumschick et al 2015; Courchamp et al. 2017; Pauchard et al 2018). In fact, Frank (2021) appears to agree with this assertion since he stated that “ecologists’ ability to precisely predict the course and impact of invasions before they occur is limited.” And yet, despite this, Frank et al. (2019) accused Guiaşu and Tindale (2018) of “mining a single invasion biology study’s abstract for a quote expressing the need for further research on tools to predict invasions and their impacts.” This characterization by Frank et al. (2019) minimizes an important on-going problem in invasion biology. First of all, Guiaşu and Tindale (2018) gave an accurate quote from the Kumschick et al. (2015) study, in the proper context. This was a fairly extensive quote which included the opening two lines of the total eight lines of the article’s abstract, which generally summarizes the main findings of a study. Secondly, this quote was much more definitive than the interpretation by Frank et al. (2019) would suggest. Kumschick et al. (2015) specifically stated that “invasion science still lacks the capacity to accurately predict the impacts” of what they called “alien species.” This was a major study with 19 authors, including some prominent invasion biologists, and it was published in an influential journal. Guiaşu and Tindale (2018) used this quote and this study as an example. As can be seen earlier in this paragraph, it is relatively easy to come up with several additional similar examples of articles which acknowledge the challenges associated with predicting the impacts of non-native species. These articles were written by leading invasion biologists, including Simberloff (2009).

If species presumed to be non-native arrive at new locations and do not have any negative effects, and since invasion biologists seem to agree that the vast majority of non-native species do not have negative effects overall, the likelihood of ecological and economic problems due to those species should be low. Therefore, advocating the eradication of such species as soon as possible after their arrival, as Simberloff has repeatedly done (for example, in 2003b and 2014), is clear evidence of a hostile attitude towards non-native species in general, including the many harmless ones. The implication of such a drastic approach is that newly introduced species do not deserve the opportunity to contribute to ecosystems, and the assumption is that the impact of non-native species has to be, or is likely to be, negative, even if we have no evidence of this, thereby justifying the application of the “precautionary principle”. There is an implicit bias in this approach, since invasion biologists seem to assume that the introduction of new species should be viewed from a negative perspective, as a source of potential problems. What if a newly introduced species may have a positive net effect on native species and the environment? And what if such positive effects may grow over time, or may become apparent years after the introduction? The immediate or early “precautionary” eradication programs suggested by some invasion biologists would prevent us from finding out if non-native species can enhance, enrich, and improve a particular ecosystem.

Instead of coming to terms with the obvious implications of their version of the “precautionary principle”, and acknowledging the potential problems with this “guilty until proven innocent” approach to non-native species, some invasion biologists and their allies prefer to just repetitively accuse their critics of “misrepresentation” and “cherry-picking”. Guiaşu and Tindale (2018) simply analyzed some of the logical inconsistencies and contradictions in the current approach of certain leading invasion biologists to non-native species. Pointing out these logical inconsistencies is not a “misrepresentation” of invasion biology, but an accurate, although justifiably unflattering, portrayal of persistent problems in this relatively new field. Granted, a shift in language from speaking of “guilty until proven innocent” to advancing a precautionary principle is welcome. After all, who would not recommend taking precautions? But if the underlying strategy of reasoning remains the same, then the shift is merely semantic and not conceptual. This was part of what concerned Guiaşu and Tindale (2018), and nothing has been said in the subsequent discussion to mitigate that concern.

In any case, it should be possible to disagree with invasion biologists about the meaning, wisdom, and usefulness of the “guilty until proven innocent”, or “precautionary”, principle, as applied to non-native species, without having to face accusations of “science denialism”—an accusation Frank (2021), for example, directed at the Guiaşu and Tindale (2018) study. After all, the so-called “precautionary principle” is ultimately based on speculation about things that may or may not happen possibly decades or centuries from now. And this “principle” could easily be extended to include native species as well. A more general analysis of some of the problems associated with the precautionary principle is offered in the next section of this article.

There is, moreover, a certain irony in seeing the charge of “science denialism” delivered against scholars who have raised concerns about a “guilty until proven innocent” principle. Among the common factors recognized by those who study science denialism is “setting impossible expectations for what science can achieve” (McIntyre 2021; see also Diethelm and McKee 2009; Cook 2010). Insofar as the demand is to keep a species out until it is known to be safe (Ruesink et al. 1995), what looks like a requirement for acceptance is no such requirement at all. Instead, on its own terms, it is a condition that can never be met, i.e., an impossible expectation.

Furthermore, determining whether the overall effect of a species on an ecosystem, a habitat, or other species is negative or not is often a highly subjective exercise (Schlaepfer et al. 2011; Guiaşu, 2016; Boltovskoy et al. 2022), which depends to a great extent on which particular ecosystem functions, habitat features, interspecific interactions, and time frames researchers choose to focus on.

In fact, invasion biologists do not even recognize non-native species as a legitimate part of the biodiversity of ecosystems into which these species have been introduced, in some cases centuries ago, and therefore refuse to include such species in biodiversity inventories and biodiversity indices (Guiaşu 2016; Schlaepfer 2018). For instance, a group of 26 authors, including Simberloff, explicitly argued that non-native species should not be included together with native species in biodiversity indices, because, in their view, this would “inflate biodiversity estimates” and also because “some non-native species can become invasive” at some point. These authors also admitted that “we still cannot be certain which non-native species will be most detrimental” (Pauchard et al. 2018). So, again, this amounts to clear discrimination against all non-native species due to their presumed biogeographic origins. How can one argue that invasion biologists do not have a generally negative view of non-native species, when such biologists refuse to even recognize non-native species as a genuine and integral part of the ecosystems where these species were introduced?

### The controversial precautionary principle and non-native species

As discussed in the previous section, the precautionary principle is frequently invoked by some invasion biologists as a justification for controlling or eliminating, or preventing the introduction of, non-native species (for example, in Simberloff 2005, 2009; Vitule et al. 2009; Simberloff et al. 2013). However, the precautionary principle is controversial and the usefulness of this principle for dealing with a variety of environmental and biodiversity-related issues has been questioned (Foster et al. 2000; Sunstein 2005; Powell 2010; Newman et al. 2017).

The precautionary principle has many formulations and can be interpreted in a variety of ways, which can lead to confusion (Foster et al. 2000; Sunstein 2005; Sandin 2007; Newman et al. 2017). For example, according to the Rio Declaration on Environment and Development (1992), “the precautionary approach” should be used “in order to protect the environment” when “there are threats of serious or irreversible damage” and, in such cases, “lack of full scientific certainty shall not be used as a reason for postponing cost-effective measures to prevent environmental degradation”. Simberloff (2003a, 2005; b) has referred to the Rio Declaration (1992) and the related 1992 Rio Convention on Biological Diversity while discussing the application of the precautionary principle to invasion biology and the control of invasive species. One of the problems is that in many cases it is difficult, if not impossible, to accurately estimate, or reasonably anticipate, what the costs associated with a newly introduced species may be, or even if that species may cause any measurable ecological harm at all. Furthermore, the notion of “harm” can be quite subjective in invasion biology, and, as discussed in a previous section, at least in some cases, this notion may be influenced more by the perception of the species as non-native, rather than by an unbiased appraisal of its actual ecological impact. This approach also ignores the potential benefits associated with the arrival of new species. In any event, it is obviously impossible to assess what the cost-effective measures for getting rid of a species would be, if we don’t know what the impact of that species will be, or even if attempting to remove the species makes any sense, from both a practical and a logical standpoint. Sometimes, control and eradication programs against non-native species can be expensive failures and can also have unintended negative consequences for native species and the environment. In such cases, the prudent approach would clearly be not to take such potentially destructive actions, especially in the absence of adequate data that would justify such a drastic course of action (Guiaşu 2016). Anticipating “serious or irreversible damage” when new species are introduced may often be unwarranted, and any species will likely bring some changes to the environment, simply by existing in that environment, in most cases without actually causing “damage”, which can also be a subjective term, influenced by our perception of the species’ biogeographic origin.

Furthermore, funds allocated for control and eradication programs against non-native species may be better spent for other, perhaps more useful, conservation or environment-related initiatives. In other words, in the absence of relevant information, the precautionary principle can be used to justify both taking action and not taking action against a particular species, so this would not seem to be a particularly useful guide for dealing with non-native species in general.

In a response to the invocation of the precautionary principle by Vitule et al. (2009) to justify an overall approach hostile to non-native fish introductions, Gozlan (2009) stated that “considering all fish introductions to have the same level of risk is counter productive as it does not engage with the process of risk assessment”. Gozlan (2009) also mentioned that Vitule et al. (2009) “make a plea for our ignorance to be the basis of ‘a precautionary approach’, using non-native fish introduction as a scapegoat.”

Larson et al. (2013) advised taking a more cautious approach to biological invasions, and stated that “when uncertainties are high, it will be unclear whether action—or inaction—will have net positive or negative consequences.” These authors added that when it comes to invasive species, “there is often little agreement about what sort of management would be appropriate.”

However, in their response to Larson et al. (2013), Simberloff et al. (2013) took the position that “there is nothing inherently cautious about doing nothing in the face of a small patch of a recently introduced plant or a pond with a population of an introduced fish” and continued by defending aggressive actions against such introduced species because, in their view, “failure to act could conceivably lead to a very bad consequence, even if the likelihood is low or the probability uncertain. This is the very spirit of the precautionary principle.” So, Simberloff et al. (2013) clearly interpret the precautionary principle as involving taking aggressive actions against non-native species, even if such species do not have proven negative effects and are very unlikely to ever have negative effects.

In a devastating critique of the precautionary principle as it is applied to the preservation of biodiversity, Newman et al. (2017) concluded that this principle may be useful “as a slogan” but, “as a principle for forming policy it is woefully problematic and not a reasonable substitute for either cost–benefit analysis or ecological risk assessment”. The authors added that this principle “promotes the idea that we can make wise decisions when we lack the necessary information to do so”, “leads to logical contradictions”, and “is so completely lacking in conceptual clarity that it is next to useless as a policy guide” (Newman et al. 2017).

Sunstein (2005) found the precautionary principle to be “literally incoherent” and “paralyzing”, since “it forbids the very steps that it requires”. The author added that “because risks are on all sides, the Precautionary Principle forbids action, inaction, and everything in between.” Sunstein (2005) also suggested that the use of the precautionary principle as a guide or a regulatory tool is facilitated by an exaggerated estimation of risk and the spread of social fear, and concluded that this principle “often operates when a moral panic is occurring.”

Larson (2011) criticized what he called the “advocacy by fear” used by some invasion biologists, and focused on particular alarmist metaphors, such as “invasional meltdown”, which was introduced by Simberloff and Von Holle (1999) “before it had full empirical support.” Simberloff et al. (2013) also found that “the spread of invasive species is, in fact, reminiscent of armies moving”. Presumably, the use of such metaphors and militaristic terms in invasion biology, which can be traced back all the way to Charles Elton’s (1958) book about invasive animals and plants, is meant to create and promote anti-non-native species attitudes among members of the general public. However, in the long run, alarmist messages and metaphors can weaken the public’s trust in scientists and scientific objectivity (Larson 2011; Guiaşu 2016). As Larson (2011) mentioned, “the problem in this context is that scientists are presenting value-laden statements as scientific facts”, which can be interpreted as a misleading and even manipulative approach.

### The strength of the “consensus” about aspects of invasion biology

Frank et al. (2019) also accuse Guiaşu and Tindale (2018) of “casting doubt on the level of scientific consensus among invasion biologists”. Some invasion biologists (for example, Russell and Blackburn 2017; Ricciardi and Ryan 2018a; Cuthbert et al. 2020) repeatedly invoke the notion of “scientific consensus” in their field to dismiss their critics. The implication is that this consensus is strong and cannot be legitimately questioned.

Frank et al. (2019) then state that: “To provide evidence of a lack of consensus in invasion biology, Guiaşu and Tindale cherry-pick results from Young and Larson’s (2011) survey of reviewers for the journal *Biological Invasions*.” In fact, Guiaşu and Tindale (2018) accurately reported relevant information from the Young and Larson (2011) study. According to this study, invasion biologists were almost equally divided on the role of non-native species in ecosystems, and 34% of these invasion biologists disagreed with the statement that “exotics are an unnatural, undesirable component of the biota and environment”, while 37% of the invasion biologists surveyed agreed with that statement. In addition, 37% of the invasion biologists participating in the survey agreed that the “invasive” label should not be linked to negative environmental impacts. This clearly suggests significant disagreement in the field, as correctly indicated by Guiaşu and Tindale (2018). Even Frank et al. (2019) appear to agree, since they admit that: “Guiaşu and Tindale rightly point out that results suggest significant disagreements in the field.” So, after criticizing Guiaşu and Tindale (2018) for accurately citing results from the Young and Larson (2011) survey to question the so-called “consensus” in invasion biology, Frank et al. (2019) agree that these results actually do indicate a lack of consensus in this field, for example on an issue as important as whether non-native species should be regarded as a natural and desirable component of biota and ecosystems or not. Frank et al. (2019) then accuse Guiaşu and Tindale (2018) of not mentioning “areas of significant agreement” among the invasion biologists surveyed by Young and Larson (2011). It is not surprising that many invasion biologists actually agree on some major issues related to their field. What is surprising, and what challenges the notion of consensus among these biologists, is the fact that there was so much disagreement on a fundamental issue—an issue central to the field—even among a large number of reviewers for the flagship journal of invasion biology. Presumably, the vast majority of these reviewers were not critics of invasion biology.

More recently, Gbedomon et al. (2020) conducted a survey of 314 individuals—about half of them biologists and half of them social or environmental scientists, to determine opinions on the perceived value of, and threats associated with, non-native species. The authors of the study found a variety of views regarding non-native species within the scientific community. A majority of the respondents to this survey agreed that biodiversity should include all species—native and non-native. A majority of the scientists surveyed also agreed that the “measurement of the impact of invasive species should be based on the net biological, social, and economic effects”, thus taking into account the positive effects as well and not just the negative ones. Gbedomon et al. (2020) considered this finding to be “a marked departure from current methods that focus only on the adverse effects of a subset of non-native species considered as invasive.” Boltovskoy et al. (2022) also found that the estimates of the economic impacts of invasive species are flawed and misleading, since such estimates typically include only the costs associated with such species, but not the benefits. Furthermore, these costs are often very difficult to estimate accurately (Guiaşu 2016; Boltovskoy et al. 2022).

Therefore, it does appear that the “consensus” about non-native species invoked by some leading invasion biologists may not be as strong as they claim.

## The olive branch?

We appreciate the attempt to understand alternative points of view about non-native species, and the more positive overall approach, taken by Frank et al. (2019), at least in parts of the section titled “Reasonable debates about invasive species”, towards the end of their article. We are somewhat encouraged by this more nuanced perspective and the relatively civilized nature of this particular debate, although, in general, the gap between invasion biologists and their critics remains large, and may, in fact, be widening (Warren 2021). We would have found this more conciliatory approach by Frank et al. (2019) even more convincing, however, if Frank (2021) did not accuse Guiaşu and Tindale (2018) of science denialism, an accusation we find unfortunate and unwarranted, as discussed earlier in this article.

The more measured and careful overall response by Frank et al. (2019) is in sharp contrast to some recent attacks by leading invasion biologists on critics of aspects of invasion biology. For example, Russell and Blackburn (2017) and Ricciardi and Ryan (2018a) accused critics of invasion biology of “science denialism” and questioned the motivation and good faith of these critics. Furthermore, Russell and Blackburn (2017) compared critics of invasion biology to those who question the cause of AIDS, or the negative effects of smoking, or the evidence for evolution, while Ricciardi and Ryan (2018a) compiled an eclectic and controversial list of sources which, in their view, showed evidence of science denialism. This list included peer-reviewed articles by respected ecologists whose views on non-native species Ricciardi and Ryan (2018a) disagreed with. Even more recently, in an article with 19 authors, including Simberloff, Cuthbert et al. (2020) went as far as to “urge journal editors to reconsider acceptance of denialist essays”, which is essentially a call for censorship. This is akin to one team asking the referee to disqualify the opposing team before the contest (or, in this case, the debate) can begin. It is always easier to win by default, when the other side is not allowed to make its case. These types of articles are indicative of the increasingly intransigent, intolerant and dismissive approach taken by some invasion biologists towards critics of their field.

It could be argued that articles such as the ones by Russell and Blackburn (2017) and Ricciardi and Ryan (2018a) also promote a “guilt by association” fallacy, by vaguely connecting researchers whose views on non-native species these authors dislike with individuals who reject evolution or deny the negative effects of smoking and so on, and presenting no clear and specific evidence in support of these overly broad and damaging assertions.

Not surprisingly, there was strong pushback by a variety of ecologists and other researchers (for example, Crowley et al. 2017; Davis and Chew 2017; Tassin et al. 2017; Boltovskoy et al. 2018; Munro et al. 2019 and others) against these types of accusations.

Based on these recent exchanges in the scientific literature on non-native species, we see no particular reason to be optimistic, in general, that the opposing sides in this debate are getting closer to reaching a common ground at this time.

## Conclusions and implications

The problems associated with invasion biology identified by Guiaşu and Tindale (2018) remain. The crucial concept of native range is still poorly defined, and uncertainties persist with respect to the geographic origins, the extent and history of the native ranges, the means of dispersal, and the ecological and economic impact of numerous species in various parts of the world. Species are still labelled as “invasive” and “damaging” too casually and subjectively, in quite a few cases, and control and eradication programs are undertaken sometimes without adequate supporting evidence. Unfortunately, too often, instead of reflecting on these fundamental problems and trying to address them, or at least acknowledge them in a straightforward manner, some leading invasion biologists choose to blame the messengers, or their critics, instead, for bringing up these problems in relevant publications. This approach is unlikely to lead to any genuine progress or more accountability in this field.

Humans have dramatically changed the distributions of numerous species for thousands of years, and deciding what is a “natural” range or trying to recreate supposedly “pristine” ecosystems and return them to a mythical state that may have existed prior to human influences are unrealistic goals in a rapidly changing and increasingly cosmopolitan world dominated by novel ecosystems which include intricately intertwined assemblages of both native and non-native species (Boivin et al. 2016; Guiaşu 2016; Warren 2021).

Warren (2021) included the Guiaşu and Tindale (2018) article among the publications which promote a more inclusive vision of non-native species and a more cosmopolitan approach which is “realistic and even optimistic, replacing a loss-only outlook with an appreciation of the benefits as well as the challenges of the novel, emerging world.” We agree with this assessment, and we believe that such a more open-minded approach towards non-native species and the novel ecosystems they are an essential part of is also more pragmatic and leads to a more balanced view of the natural world and a better allocation of conservation resources.
